# Biopsychological traits of Sasang typology based on Sasang personality questionnaire and body mass index

**DOI:** 10.1186/1472-6882-14-315

**Published:** 2014-08-26

**Authors:** Soo Jin Lee, Soo Hyun Park, C Robert Cloninger, Yun Hee Kim, Minwoo Hwang, Han Chae

**Affiliations:** Department of Psychiatry, School of Medicine, Washington University, Saint Louis, MO 63110 USA; Department of Psychotherapy, Nursing and Public Health, Kyungil University, Daegu, 712-701 Korea; Department of Psychology, Yonsei University, Seoul, 120-749 Korea; Department of Sasang Constitutional Medicine, College of Oriental Medicine, Daejeon University, Daejeon, 300-716 Korea; Department of Sasang Constitutional Medicine, College of Korean Medicine, KyungHee University, Seoul, 130-701 Korea; Division of Longevity and Biofunctional Medicine, School of Korean Medicine, Pusan National University, Pusan, 609-735 Korea

**Keywords:** Biopsychological traits, Sasang typology, Sasang personality questionnaire, Body mass index

## Abstract

**Background:**

The purpose of present study was to examine biological and psychological characteristics of people according to the Sasang typology, which is popular in Korea. We evaluated the Sasang Personality Questionnaire (SPQ) as a measure of temperament, and Body Mass Index (BMI) as a measure of the somatic properties of each Sasang type.

**Methods:**

Subjects were 2506 (877 males, 1629 females) outpatients between the ages of 20 through 70 who requested traditional medical assessment and treatment in Korea. The structural validity of the SPQ was examined and its correlation with BMI was analyzed. The SPQ and BMI measures of each Sasang type across age and gender were presented and their differences were analyzed with Analysis of Variance.

**Results:**

Confirmatory factor analysis and path analysis identified an acceptable three-factor structure of the SPQ measuring differences in individual’s behavior, emotion, and cognition. SPQ scores (29.71 ± 1.00, 28.29 ± 0.19 and 26.14 ± 0.22) and BMI scores (22.92 ± 0.09, 25.56 ± 0.10 and 21.44 ± 0.10) were significantly (p < 0.001) different among So-Yang, Tae-Eum and So-Eum Sasang types, respectively.

**Conclusions:**

The results showed that the SPQ and BMI is a reliable measure for quantifying the biopsychological characteristics of each types, and useful for guiding personalized and type-specific treatment with medical herbs and acupuncture.

## Background

Personalized medicine tailors diagnosis and treatment to the particular characteristics of each person in order to enhance safety and treatment efficacy [1, 2]. Recently the Human Genome Project has encouraged efforts to personalize medical treatment, just as medical practitioners since antiquity have tried to personalize their diagnosis and treatment in a variety of ways, such as theories of temperament based on four humors of Hippocrates and Galen in the West, and medical typologies based on the Five Phase or Yin-Yang theories in the East [1, 3, 4].

Traditional classification systems for personalized medicine are widely used in many countries in the form of Ayurveda in India, naturopathic medical systems of body type in the Americas and Europe, constitutional medicine in China, Ikkando medicine in Japan, and Sasang typology in Korea. These contemporary traditional systems of medical classification consider the person as a whole, rather than focusing on particular organs or biological systems as is done in allopathic medicine in the West. The balance and integration of biopsychosocial functions is considered to be essential to prevent disease and to restore health in traditional approaches to person-centered medicine [3, 5]. If there were reliable ways to assess such traditional typologies, it might be possible for there to be a meaningful exchange of knowledge between holistic person-centered approaches and organ-focused approaches.

**Figure 1:**
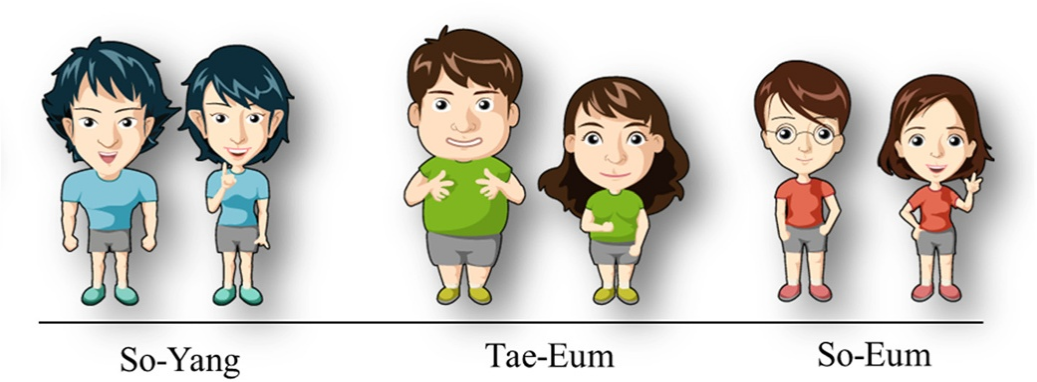
**Schematic psychobiological features of each Sasang type group.**

The Sasang typology is a classification scheme in traditional Korean medicine that is based on more than a thousand years of clinical experience. It co-evolved along with the Yin-Yang and Confucian traditions in Korea, and was systematized by Lee Je-ma in his book *Longevity and Life Preservation in Oriental Medicine* in 1894 [6]. According to the Sasang typology, it is helpful to personalize traditional medical treatment by distinguishing people into four types based on the maturity and stability of Yang (i.e., the active principle of life associated with men, creative change, heat, and light) or Yin (i.e., the passive principle of life associated with women, persistence, cold, and darkness). Specifically, the four Sasang types are called Tae-Yang (TY, “Big Yang”, who are generally stable and active), So-Yang (SY, “Little Yang”, who are generally unstable and active), Tae-Eum (TE, “Big Yin”, who are generally stable and passive”), and So-Eum (SE, “Little Yin”, who are generally unstable and passive) [7]. These distinctions between Sasang types are the basis for type-specific prevention, treatment, and rehabilitation procedures using medical herbs and acupuncture combined with appropriate alterations in lifestyle [2, 4, 7–10]. The original description [6] and results of previous research on biopsychological characteristics [2, 7, 11], pathophysiology [12, 13], and interventions [9, 10] based on Sasang typology as well as illustrative features of each Sasang types are summarized in Table 1 and Figure 1.

**Table 1:** **Review of characteristics in Sasang typology**

Sasang type (prevalence)	Tae-Yang (太陽) (<0.1%)	So-Yang (少陽) (20%)	Tae-Eum (太陰) (50%)	So-Eum (少陰) (30%)
Origin of the nature	Sorrow (哀) by benevolence (仁)	Anger (怒) by righteousness (義)	Gladness (喜) by courtesy (禮)	Enjoyment (樂) by wisdom (智)
	They feel sad when they realize their self-transcendent idea is obstructed.	They become angry when they are blocked. The anger can be regulated by fairness.	Social approval can be obtained with courtesy. They are glad when they get what they want.	Worries can be relieved with wisdom. They enjoy what they have now.
Personality or temperament	Masculine, move forward, original	Active, externally oriented, talented for business.	Feminine, withdrawn, conservative.	Still, internally oriented, self-directed.
	Independent, creative, positive, persistent, progressive, charismatic, disinhibited.	Unstable, easily get bored, sacrificing, righteous, easily acceptable, quick tempered, active, easy-going.	Gentle, commercial, warm, endurable, humorous, look foolish, reflective, social, hospitable, coward.	Neat, mild, negative, intelligent, organized, patient, jealous, perseverant, passive, static, meticulous.
	Rash mind (急迫之心)	Anxious mind (懼心)	Afraid or fearful mind (怯心)	Nervous mind. (不安定之心)
		High Extraversion and low Neuroticism (NEO-PI). High Novelty-Seeking and low Harm-Avoidance (TCI). High SPQ	In the middle of So-Yang and So-Eum as for the Extraversion, Novelty-Seeking, Harm-Avoidance and SPQ. High Positive Affect (PANAS). Low in Trait Anxiety (STAI).	Low Extraversion and high Neuroticism. Low Novelty-Seeking and high Harm-Avoidance. Low SPQ. Low Positive Affect. High in Trait Anxiety.
Body shape or constitution	Developed nape of the neck, slender waist	Developed chest, small hip	Thick waist, weak nape of the neck	Developed hip, weak chest
		Short and little	Tall and big	Short and little
		Similar to the So-Eum type, but less small and slim than So-Eum type	High BMI, BMR, body fat mass, bone density and Waist-hip ratio. High width-height ratio of face. Bigger neck and chest circumference	Low BMI and Waist-hip ratio. Low width-height ratio of face. Smaller neck and chest circumference
Pathophysiological characteristics	Large lung system, small liver system (肺大肝小)	Large spleen/stomach system, small kidney system (脾大腎小)	Large liver system, small lung system (肝大肺小)	Large kidney system, small spleen/stomach system (腎大脾小)
	Strong sympathetic activation, weak anabolism and energy-storing	Strong intake and digestion, weak waste discharge	Strong anabolism and energy-storing, weak sympathetic activation	Strong waste discharge, weak intake and digestion
			High SDFI and low FDQOL. High insulin resistance. high Triglyceride, cholesterol concentration, and blood pressure	Low SDFI and high FDQOL. Low immune function
Concerns for the good health	Enough urination	Easy with defecation	Enough perspiration	Good digestion
	Avoid dehydration and overexertion of mental and bodily resources	Avoid over-activation and overloads of bodily functions	Maintain adequate level of catabolic, sympathetic and circulatory system	Maintain healthy digestive function, peristalsis, and body heat
Frequent symptoms or disease	Emesis, nervousness/neurasthenia, sudden weakness in lower extremities	Constipation, gastroesophageal (laryngopharyngeal) reflux disease, affective disorder, insomnia, heat on chest	No perspiration, diabetes, metabolic syndrome, hypertension, stroke, obesity, obstructive sleep apnea, irritable bowel syndrome	Indigestion or dyspepsia, upper respiratory infection, neurotic symptoms
type-specific medical herbs	Chaenomelis Fructus, Acanthopanacis Cortex, Phragmitis Rihizoma	Rehmanniae Radix, Corni Fructus, Hoeoen, Alismatis Rhizoma, Osterici Radix, Angelicae Pubescentis Radix	Ephedrae Herba, Liriopis Tuber, Schisandrae Fructus, Dioscoreae Rhizoma, Platycodi Radix, Coicis Semen, Puerariae Radix	Ginseng Radix, Atractylodis Rhizoma Alba, Glycyrrhizae Radix, Cinnamomi Cortex, Citri Pericarpium, Zingiberis Rhizoma Crudus
type-specific acupuncture points	Diagnosis with HT8. Treatment with LR3(+)/ LU9(−)	Diagnosis with HT3. Treatment with HT7(+)/ SP3(−)	Diagnosis with HT4. Treatment with LU9(+)/ LR3(−)	Diagnosis with HT7. Treatment with SP3(+)/ LI4(−)

TCI, Temperament and Character Inventory; NEO-PI, NEO Personality Inventory; SPQ, Sasang Personality Questionnaire; PANAS, Positive and Negative Affect Schedule; STAI, State and Trait Anxiety Inventory; SDFI, Sasang Digestive Function Inventory; FDQOL, Functional Dyspepsia-Related Quality of Life; BMI, Body Mass Index.

To briefly summarize, the So-Yang type is an active, extroverted, inquisitive, outgoing, quick-tempered, excitable, dynamic, easy-going, and impulsive person with strong interest in the outside world, while the So-Eum type is introverted, organized, reserved, patient, negative, cautious, passive, static, meticulous and nervous person focused on his/her inner world. Although the Tae-Eum type lies in between the So-Yang and So-Eum type in regards to their psychological features, people with the Tae-Eum type have a higher body mass index and bigger chest circumference compared to the So-Eum and the So-Yang type. The Tae-Yang type is an originative, independent, charismatic and creative person focusing on achievement compared to the Tae-Eum type who is more likely to be conservative, withdrawn, warm, adaptable, reflective, positive, sensitive to others and endurable and preferring stability.

There have been many and diverse studies on Sasang type-specific psychological [4, 7, 11], physical [2, 12, 14, 15], pathophysiological [12, 13, 16] and genetic [1, 17] characteristics. These studies were carried out to rapidly recognize and understand underlying mechanisms of disease in order to treat patients effectively in clinical settings. One systematic review of extant research literature associated with Sasang typology identified two super-factors of Extraversion (i.e., active versus passive) and Neuroticism (i.e., neurotic versus stable) as the major domains [7, 18] in terms of psychological or temperamental characteristics. The Sasang Personality Questionnaire (SPQ) was recently developed for the objective dimensional measurement of such psychological characteristics and has shown clinical validity and reliable psychometric properties [4, 11, 19].

The SPQ has three subscales including SPQ-Behavior, SPQ-Emotionality, and SPQ-Cognition. It is positively correlated with Novelty Seeking (i.e., impulsive, disorderly versus rigid, orderly) of Cloninger’s Temperament and Character Inventory (TCI) and Extraversion of NEO Personality Inventory (NEO-PI), and negatively correlated with Harm Avoidance (i.e., anxious, shy versus risk-taking, outgoing) on the TCI in clinical setting with child and adult sample [9, 20]. The SPQ score of the So-Eum, Tae-Eum, and So-Yang Sasang types were significantly different from each other, with the rank order of SPQ scores being SE < TE < SY [4, 11, 19]. The fourth Tae-Yang type was too infrequent to evaluate in samples of the general population.

Furthermore, the body shapes or constitutional characteristics of Sasang typology have been examined through measures such as the Body Mass Index (BMI), Body Fat Mass [2, 12], and circumference of the neck and chest [14], with the rank order of these physical characteristics being SE < SY < TE [13].

Together the SPQ and BMI may be useful to identify people with different Sasang types reliably, thereby helping in clinical assessment and treatment [13, 19]. However, there have been few studies to examine such biopsychological characteristics along with variation in age and gender.

Even the *Longevity and Life Preservation in Oriental Medicine*[6], the original book of Je-ma Lee who is the founder of Sasang typology, briefly described the difference in biopsychological characteristics across gender and age [7, 18]. Studies measuring temperamental and constitutional characteristics in clinical samples were performed more recently [9, 20, 21]. Such studies of biopsychological features across the life span provide a fundamental and pivotal basis for future clinical research and practice in Sasang typology [20].

To this end, the present study was conducted on a large-scale nationwide clinical sample to examine the biopsychological characteristics of each Sasang types using the SPQ and BMI after reconfirming the factor structure of the SPQ. Results from this study would be used for the standardization of biopsychological characteristics across gender and age for classification according to the Sasang typology, thereby providing a foundation for Sasang type identification and personalized type-specific interventions.

## Methods

### Subjects

The present study used the data pertaining to Sasang type identification and intervention from the Korea Constitution Multicenter Bank, which has acquired written informed consent from the participants. Sasang type of subjects was determined by certified clinical specialists as described in previous research [9].

Subjects included in the database were outpatients who visited a hospital for Korean Oriental Medicine between 2007 and 2010 in major cities of Korea including Gwangju, Jeonju, Daejeon, Daegu, Pusan, and Seoul. These participants met all of the following inclusion criteria: (i) made a minimum of five outpatient visits to the certified Sasang typology specialist who made the type classification considering biopsychological features and pathophysiological symptoms, (ii) underwent pharmacological management prescribed for the particular *Sasang* type for 50 days or more, (iii) demonstrated clear improvement in their chief complaints and pre-existing symptoms and/or exhibited a unique improvement pattern and (iv) had clear documentation of medication treatment response in medical charts and did not manifest significant adverse events.

This study received approval from the Institutional Review Board of the School of Korean Medicine, Pusan University (KMED IRB 2013–3).

### Tools

#### A. Sasang Personality Questionnaire (SPQ)

Sasang Personality Questionnaire (SPQ) is a 14-item self-report assessment tool measuring temperament characteristics from the perspective of the Sasang typology. It is composed of three subscales that measure behavior (SPQ-Behavior: SPQ-B), emotion (SPQ-Emotion: SPQ-E), and cognition (SPQ-Cognition: SPQ-C) [4]. The SPQ scores of total and each subscale were found to be decreased orderly in the So-Yang, Tae-Eum, and So-Eum Sasang types. The internal consistency of the SPQ, SPQ-B, SPQ-E, and SPQ-C were 0.81, 0.74, 0.62 and 0.62, respectively.

#### B. Body Mass Index (BMI)

BMI is determined by dividing the individual’s weight (kg) by height squared (m^2^) of each participants [2]. Previous research has consistently reported that the BMI score of the Tae-Eum Sasang type is the highest, and that of the So-Eum type the lowest [12, 19].

### Statistical analysis

Descriptive statistics on gender, age, job, education, marital status and Sasang types were analyzed, and χ^2^ test was conducted to examine the differences between Sasang types across age and gender.

Path analysis and confirmatory factor analysis (CFA) were used to test the three-factor model structure of the SPQ which was established in the previous study [4]. Since the model fit of the path analysis model is affected by the sample size, χ^2^, Tucker-Lewis Index (TLI), Goodness of Fit Index (GFI), Adjusted Goodness of Fit Index (AGFI), and Root Mean Square Error of Approximation (RMSEA) were used as the fit index in this study [22].

Pearson’s correlation was calculated to examine the correlations between BMI and SPQ and its subscales. Analysis of Variance (ANOVA) and Tukey post-hoc analysis were used to examine the difference between Sasang type groups on the SPQ and BMI across gender and age.

Statistical results were presented as frequency (%) or mean ± standard error, and statistical significance level was set at p < 0.05, p < 0.01, and p < 0.001. PASW Statistics 18.0 (IBM, Armonk, NY) was used for all statistical analysis.

## Results

### Demographic data

A nation-wide sample of 2565 individuals between the ages of 20 and 79 (mean age = 49.03 ± 14.23) was included, and the demographic characteristics are presented in Table 2. The observed ratio of Tae-Yang, So-Yang, Tae-Eum, So-Eum types was 59:857:987:662. Since the number of people with the Tae-Yang type was too small for analysis [2, 6, 9], they were excluded from statistical analysis examining differences between Sasang types.

**Table 2:** **Demographic characteristics of the subjects by age**

Ages	20s	30s	40s	50s	60s	70s	Total
Gender	254(9.9)	468(18.2)	590(23.0)	600(23.4)	430(16.8)	223(8.7)	2565(100)
Male	82(9.2)	147(16.4)	207(23.2)	223(24.9)	161(18.0)	74(8.3)	894(100)
Female	172(10.3)	321(19.2)	383(22.9)	377(22.6)	269(16.1)	149(8.9)	1671(100)
Job	254(9.9)	468(18.3)	590(23.0)	600(23.4)	430(16.8)	222(8.7)	2564(100)
Managerial	1(2.0)	5(10.0)	18(36.0)	18(36.0)	7(14.0)	1(2.0)	50(100)
Professional	75(19.6)	137(35.9)	105(27.5)	52(13.6)	11(2.90)	2(0.50)	382(100)
Administrative	39(12.5)	120(38.5)	98(31.4)	49(15.7)	5(1.6)	1(0.30)	312(100)
Service	5(2.6)	33(17.2)	62(32.3)	63(32.8)	26(13.5)	3(1.6)	192(100)
Sales	4(3.5)	15(13.3)	34(30.1)	38(33.6)	18(15.9)	4(3.5)	113(100)
Agricultural	0(0.0)	2(1.3)	24(15.5)	37(23.9)	47(30.3)	45(29.0)	155(100)
Skilled trades	1(1.6)	7(11.5)	19(31.1)	25(41.0)	8(13.1)	1(1.6)	61(100)
Plant & machine operatives	1(2.3)	9(20.5)	7(15.9)	22(50.0)	5(11.4)	0(0.0)	44(100)
Elementary occupations	0(0.0)	3(7.5)	8(20.0)	17(42.5)	10(25.0)	2(5.0)	40(100)
Others	128(10.5)	137(11.3)	215(17.7)	279(23.0)	293(24.1)	163(13.4)	1215(100)
Education	254(9.9)	468(18.3)	590(23.0)	600(23.4)	429(16.7)	222(8.7)	2563(100)
None	0(0.0)	0(0.0)	5(3.5)	11(7.8)	52(36.9)	73(51.8)	141(100)
Elementary school	0(0.0)	0(0.0)	18(5.1)	112(32.0)	135(38.6)	85(24.3)	350(100)
Middle school	0(0.0)	2(0.7)	44(15.1)	127(43.6)	94(32.3)	24(8.2)	291(100)
High school	77(10.9)	98(13.9)	216(30.6)	203(28.7)	88(12.4)	25(3.5)	707(100)
College	148(18.2)	273(33.6)	226(27.8)	107(13.2)	47(5.8)	12(1.5)	813(100)
Graduate school	29(11.1)	95(36.4)	81(31.0)	40(15.3)	13(5.0)	3(1.1)	261(100)
Marital status	254(9.9)	468(18.3)	590(23.0)	598(23.3)	430(16.8)	223(8.7)	2563(100)
Single	229(56.5)	132(32.6)	26(6.4)	10(2.5)	5(1.2)	3(0.7)	405(100)
Married	25(1.2)	330(16.4)	547(27.2)	558(27.7)	383(19.0)	169(8.4)	2012(100)
Divorced	0(0.0)	6(15.8)	14(36.8)	13(34.2)	4(10.5)	1(2.6)	38(100)
Widowed	0(0.0)	0(0/0)	3(2.8)	17(15.7)	38(35.2)	50(46.3)	108(100)
Sasang type	254(9.9)	468(18.2)	590(23.0)	600(23.4)	430(16.8)	223(8.7)	2565(100)
Tae-Yang	5(8.5)	21(35.6)	15(25.4)	12(20.3)	4(6.8)	2(3.4)	59(100)
So-Yang	81(9.5)	152(17.7)	201(23.5)	207(24.2)	149(17.4)	67(7.8)	857(100)
Tae-Eum	77(7.8)	141(14.3)	226(22.9)	242(24.5)	193(19.6)	108(10.9)	987(100)
So-Eum	91(13.7)	154(23.3)	148(22.4)	139(21.0)	84(12.7)	46(6.9)	662(100)

Parenthesis shows the percentage.

After exclusion, there were 877 (35%) men in the 2506 subjects, and the mean age of the total sample was 49.15 ± 14.27. Subjects were most often in their 40-50 s (1163 subjects; 46.4%). There were no significant differences in gender composition for each age group (χ^2^ = 6.591, df = 5, p = 0.253), but there was a significant difference in Sasang type distribution by age group (χ^2^ = 54.614, df = 10, p < 0.001) and gender (χ^2^ = 15.088, df = 2, p = 0.001).

### Structural model of the SPQ

Confirmatory factor analysis on the 14-item SPQ verified the original three factors structure with Promax rotation and explained 48.57% of the total variance: SPQ-B, 29.01%, SPQ-E, 7.86%, and SPQ-C, 11.69%. As a result of model fit in path analysis, the χ^2^ of the modified three-factor model was 961.2, TLI, 0.844, GFI, 0.947, AGFI, 0.920, and RMSEA, 0.071. Although the TLI and RMSEA were relatively low, the GFI showed goodness of fit higher than the standard criteria and considering the large sample size of the present study, the fit of the model is acceptable [22].

### Correlation between BMI and SPQ and its subscales

Correlational analysis between BMI and SPQ and its subscales is shown in Table 3. The total SPQ score has relatively high correlations with SPQ subscales: SPQ-B (r = 0.841, p < .01), SPQ-C (r = 0.832, p < 0.01) and SPQ-E (r = 0.692, p < 0.01). However, BMI showed fairly low correlations with the SPQ (r = 0.121, p < .01), SPQ-B (r = 0.083, p < .05), SPQ-C (r = 0.181, p < 0.01) and SPQ-E (r = 0.014, ns).

**Table 3:** **Correlation matrix for Sasang personality questionnaire and body mass index**

	SPQ-behavior	SPQ-emotion	SPQ-cognition	BMI
SPQ	.841^**^	.692^**^	.832^**^	.121**
SPQ-behavior		.355^**^	.579^**^	.083**
SPQ-emotion			.374^**^	.014
SPQ-cognition				.181**

**p < 0.01. SPQ, Sasang Personality Questionnaire; BMI, Body Mass Index; Bold type represents correlation coefficient more than 0.4.

### SPQ and BMI characteristics of Sasang types across gender and age group

The total scores on the SPQ (29.71 ± 1.00, 28.29 ± 0.19 and 26.14 ± 0.22) and BMI (22.92 ± 0.09, 25.56 ± 0.10 and 21.44 ± 0.10) of So-Yang, Tae-Eum, and So-Eum Sasang types, respectively, were found to be significantly different (p < 0.001) among Sasang types.

**Figure 2:**
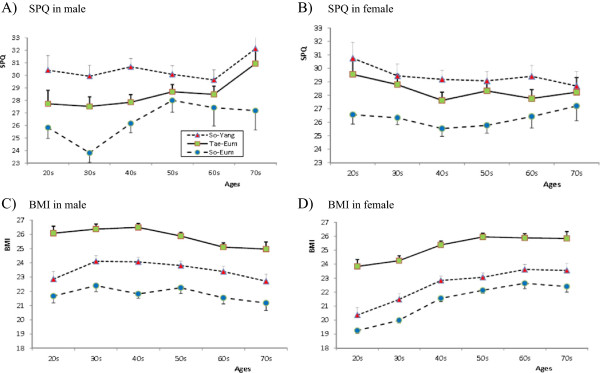
**The biopsychological traits of each Sasang types as measured by SPQ and BMI. A)** SPQ in male, **B)** SPQ in female, **C)** BMI in male, **D)** BMI in female. The So-Yang, Tae-Eum and So-Eum are represented with red triangle, green square and blue circle, respectively. The line represents the mean and the whisker represents standard error of each Sasang type groups.

ANOVA was conducted to examine differences between the Sasang types across age (Table 4) and demonstrated significant differences between types in both SPQ and BMI. Tukey post-hoc analysis showed that the SPQ score for the So-Yang group was significantly higher than that of So-Eum group (p < 0.01), and the score of BMI in Tae-Eum group was significantly higher than that of So-Eum and So-Yang group (p < 0.01). In addition, such marked distinction of each Sasang types remained after gender and age were put together for the further analysis in SPQ and BMI (Figure 2). The SPQ score of the So-Yang type group was significantly (p < 0.01) higher than that of So-Eum group except 50s, 60s and 70s of male and 70s of female, and the score of BMI in Tae-Eum group was significantly (p < 0.01) higher than that of So-Eum group regardless of gender and age.

**Table 4:** **Sasang personality questionnaire and body mass index measures of each Sasang types across ages**

	20s	30s	40s	50s	60s	70s
SPQ						
So-Yang	30.65 ± .58	29.58 ± .41	29.70 ± .42	29.42 ± .42	29.49 ± .50	30.28 ± .81
Tae-Eum	28.94 ± .61	28.32 ± .48	27.71 ± .40	28.49 ± .36	28.06 ± .45	29.01 ± .59
So-Eum	26.32 ± .55	25.56 ± .43	25.72 ± .45	26.41 ± .50	26.71 ± .74	27.20 ± .90
F-value	14.905 ***	22.451 ***	19.705 ***	11.313 ***	5.504 **	3.272 *
Tukey post-hoc	SY>SE, TE>SE	SY>SE, TE>SE	SY>TE>SE	SY>SE, TE>SE	SY>SE	SY>SE
BMI						
So-Yang	21.04 ± .25	22.24 ± .22	23.25 ± .20	23.32 ± .17	23.53 ± .20	23.16 ± .38
Tae-Eum	24.60 ± .42	25.03 ± .28	25.81 ± .19	25.92 ± .20	25.56 ± .19	25.59 ± .29
So-Eum	20.04 ± .24	20.73 ± .21	21.64 ± .19	22.17 ± .20	22.32 ± .30	22.11 ± .33
F-value	60.114 ***	85.809 ***	111.353 ***	98.371 ***	52.383 ***	28.619 ***
Tukey post-hoc	TE>SY, TE>SE	TE>SY>SE	TE>SY>SE	TE>SY>SE	TE>SY>SE	TE>SY, TE>SE

*,p < 0.05; **,p < 0.01; ***,p < 0.001. SPQ, Sasang Personality Questionnaire; BMI, Body Mass Index; SY, So-Yang; TE, Tae-Eum; SE, So-Eum. Data shown as mean ± Standard error.

## Discussion and conclusion

There have been extensive studies regarding to the Sasang type-specific biopsychosocial features, pathophysiological symptoms, and interventions with diverse expertise. However, most of the previous studies have focused on particular clinical and normal groups with limited variation in gender or age [7, 12, 16], so possible changes with age could not be identified. Therefore, the lifetime pattern of biopsychological changes of each Sasang types was explored in the present large, nationwide study using the SPQ and BMI as reliable measures of biopsychological characteristics of the Sasang typology.

As a first step, the three-factor model of SPQ was examined and found to be acceptable. The correlation analysis between SPQ and BMI showed only weak correlation, confirming that these two aspects of temperament and constitution represent largely independent biopsychological features of Sasang typology [19].

The SPQ score provided stable temperamental differences that increased in the order of So-Eum, Tae-Eum, and So-Yang Sasang type even when the gender difference was considered. The age trend of total SPQ score in each Sasang type groups was found to be relatively flat, consistent with previous findings about the stability with age of the personality traits of Extraversion [23] and of Novelty Seeking and Harm Avoidance [24]. This result is consistent with previous studies on the psychological features of Sasang typology [4, 25] that support the rank ordering of SE < TE < SY axis for the total score of SPQ.

It was observed that the SPQ tends to be low in the 30’s and high in the 50’s for men. This may be related to the level of education because 368 (78.6%) participants in their 30’s were college or graduate school graduate, whereas only 121 (24.5%) participants in their 50’s were this highly educated. In addition, with increasing age, the SPQ score for men showed an increasing trend and a decreasing trend for women. This apparent life span change may be influenced by social interaction because variation in Extraversion, Novelty Seeking, and Harm Avoidance can all be influenced by the social environment [8, 26]. However, this possibility should be examined in future studies because multiple biological, educational, and social variables may be confounded in our cross-sectional study of people of varying ages.

The rank order of BMI scores showed an increase from So-Eum, So-Yang, and Tae-Eum type, confirming the distinguishing phenotypic features of each Sasang types in previous studies [2, 12, 19]. These BMI differences between Sasang types were consistently maintained even when gender and age was considered. Such results appear to explain the clinical characteristics of people with the Tae-Eum Sasang type who often have high triglyceride and cholesterol levels [2], body fat mass [2], insulin resistance [27], and high blood pressure [28]. Furthermore, the change in BMI with age is consistent with a previous study of the general population in Korea [29], which showed BMI decreasing with age for men and increasing with age for women.

An interesting aspect of the present study is that similarities surpassing time and culture can be seen when our results are compared to other studies involving medical typologies across the world [3, 7, 30]. For instance, the three common Sasang types are similar to the Melancholic, Choleric, and Phlegmatic humoral types of Hippocrates and Galen [31], the Asthenic, Athletic, and Pyknic types of Kretschmer [32], the Ectomorph, Mesomorph, and Endomorph somatotypes of Sheldon [33], and Vata, Pitta, and Kapha of Prakriti in Ayurveda [34].

As for the psychological features, previous research [7, 9, 18] has suggested interesting similarities of various temperament typologies in western psychology with Sasang typology. For example, the sanguine, melancholic, choleric, and phlegmatic types proposed by Galen are remarkable similar to the phenotypic characteristics of Tae-Yang, So-Eum, So-Yang, and Tae-Eum Sasang types, respectively [7]. The psychological theories of Avicenna [35], Kant [36], Wundt [37], Adler [38], Pavlov, Gray [39], Eysenck [40] and Cloninger [41] were suggested to be related to the temperament types of Hippocrates and Galen [42, 43] from the perspective of the West [44].

Extraversion and Neuroticism have also been proposed as domains explaining the psychological characteristics in Sasang typology [7, 9, 18]. The relevance of these personality factors could be understood when it is recognized that the name for the four Sasang types is a theoretical combination of two terms, Eum-Yang (Yin-Yang or Passive-Active) and Tae-So (Big-Little or Large-Small), although the name itself may not explain each Sasang types in full [2].

The Big-Little (Tae-So) concept might come from the Confucianism, which was based on studies of social life. Accordingly, a Big person represents someone who has a more inclusive or bigger personal perspective on society, that is, one who is more generous, caring, forgiving, considerate and selfless as well as someone who has more fully developed higher cognitive functions, and who is thereby more stable, mature, and non-neurotic personality in general [45, 46]. In contrast, the Yin-Yang (Eum-Yang) concept originated in Taoism, which focused on explaining the rules of nature and so has served as a theoretical backbone of traditional medicine for thousands years in the East. The Yin-Yang aspect embodies the two opposing and complementary sides of nature as introvert-extrovert, cold-hot, wet-dry, moon-sun, night-day, dark-bright, femininity-masculinity, fast-slow, soft-solid, active-passive, and so on. The four possible combinations of these two dichotomies might form the basis for the Sasang typology.

Typologies of personality were originally understood in terms of their similarity to variation in seasons of the year, temperature (cold-warm), and humidity (wet-dry), as in the humoral theory of Hippocrates and Galen [39, 47, 48] and in the models of temperament and personality developed by Pavlov, Eysenck, and Gray [39, 42].

The Sasang type of a person seems to be a clinical prototype retaining biopsychological profiles that remain across the life span and help to explain clinical patterns of type-specific pathophysiological symptoms along with type-specific treatment responses [7, 10, 12]. If we are able to investigate and compare the Eastern and the Western perspectives using reliable biopsychosocial methods like the TCI [7, 20], a more integrative outlook on the human nature could be achieved [3]. The TCI is regarded as a well-established personality test measuring psychobiological processes within the person and the maturity of personality development and its implication on health as well as a person-centered and multidimensional profile of neurobiological predispositions which is considered as the foundation of contemporary traditional personalized medicine [20, 49–51].

A number of limitations of the present study should be mentioned. First, this study examined the life span biopsychological changes of each Sasang type with pooled cross-sectional measures. However, these findings should be confirmed in longitudinal studies.

Second, subjects below the age of 19 were not included in current study due to the difficulty of recruitment and absence of standardized measures for child and adolescent subjects [20]. Previous research with the Junior TCI Novelty Seeking scale of children is remarkably similar to that in adults [9], so more research is warranted in younger subjects.

Third, BMI is a well-established index for international health studies and has shown usefulness in Sasang typology studies with Asian population [2, 12–15]. However, the BMI can be used for the measure of obesity or adiposity, and the epidemic of obesity in the West may make it difficult to distinguish among somatotypes because obesity will obscure differences related to constitutional typologies. We suggest that the circumference ength of neck or chest [14], Bitragus to Submandibular arc length [52], height-width ratio of face [15], basal metabolic rate [53], and Ponderal Index may be more useful alternatives for the further anthropometric studies.

Last but not the least, the Tae-Yang type subjects were not included in the Sasang type group comparison due to their small number (n = 59). Such infrequency has been observed in many previous studies in which the proportion of that group is supposed to be less than 0.1% [2]. However, the SPQ and BMI of the entire Tae-Yang type group was 29.29 ± 0.80 and 21.15 ± 0.36, respectively, which may be considered that the Tae-Yang type would be physically akin to the So-Eum type [12], while psychologically to the So-Yang type. The biopsychological features of Tae-Yang type with objective measures should be needed for the future studies.

## Conclusions

In conclusion, the present study provided standardized biopsychological characteristics of the Sasang types using SPQ and BMI. We demonstrated stable patterns of psychobiological characteristics for each Sasang type despite gender and age in a nationwide sample for the first time. This study provides a reliable quantitative measure for the Sasang typology as a useful guide to effective personalized medical intervention.
